# Transphyseal Curettage of Epiphyseal Brodie’s Abscess: A Case Report

**DOI:** 10.7759/cureus.73221

**Published:** 2024-11-07

**Authors:** Ahmed H Elhessy, Mohamed Said, Amr Abdelgawad

**Affiliations:** 1 Orthopedic Surgery, Nemours Children's Hospital, Jacksonville, USA; 2 Medicine, State University of New York (SUNY) Downstate Health Sciences University, New York, USA; 3 Orthopedics, Maimonides Medical Center, Brooklyn, USA

**Keywords:** brodie‘s abscess, epiphyseal, growth arrest, subacute osteomyelitis, transphyseal curettage

## Abstract

Epiphyseal Brodie's abscesses represent a rare, slow-progressing form of osteomyelitis that contrasts with the more aggressive types of infection typically seen in bone. These abscesses develop from a low-grade infection and progress gradually, posing unique challenges for treatment due to their proximity to the growth plate and joint structures. While the literature on managing epiphyseal Brodie's abscesses is limited, common treatments include antibiotics and surgical drainage. However, removing these lesions can be complicated by the risk of growth plate damage.

This case report describes a patient with an epiphyseal Brodie's abscess that persisted despite standard curettage. We employed an acute-angle trans-physical curettage technique, which allowed for complete lesion excision while preserving the integrity of the growth plate and minimizing the risk of growth disturbance. By accessing the abscess through the diaphysis, metaphysis, and physis, this method proved to be an effective treatment option for Brodie's abscesses located near the physis.

## Introduction

Brodie's abscess is a subacute or chronic osteomyelitis of the bone that occurs in children and adolescents [1-3]. It is named after Sir Benjamin Brodie, an English surgeon who first described the condition in the early 19th century. Unlike other types of osteomyelitis that can spread quickly and cause significant damage to bone and surrounding tissue, Brodie's abscess develops from a low-grade infection that progresses slowly over time and may not produce any obvious symptoms for weeks or months [4-6]. Brodie's abscess is more common in boys and is usually caused by a bacterial infection (usually *Staphylococcus aureus*) that reaches the bone via the bloodstream [7,8]. The abscess location is mostly metaphyseal; however, sporadic cases were affected in the epiphysis [9,10]. Epiphyseal Brodie's abscess treatment typically involves a combination of antibiotics and surgical drainage of the infected area [10-12].

Epiphyseal Brodie's abscess symptoms may vary but can include localized pain, swelling, and tenderness in the affected area/joint. Epiphyseal Brodie’s abscess can also cause limitation of the adjacent joint movement. Treatment for epiphyseal Brodie's abscess is not commonly discussed in the literature, yet it typically involves a combination of antibiotics and surgical drainage of the infected area [11,12]. Without proper treatment, the infection can cause significant damage to the bone and surrounding tissue and may lead to long-term complications such as bone deformities and growth disturbances [13].

In this article, we describe a case of epiphyseal Brodie’s abscess that was successfully treated with surgical debridement by transphyseal approach, entering the lesion from the diaphysis, then the metaphysis, then the physis, and finally the lesion by an acute angle.

## Case presentation

A 10-year-old boy presented with his parents to the clinic with a few months of insidious onset left knee pain. Examination revealed minimal effusion in the left knee, accompanied by a painful restriction in the final degrees of flexion. The patient exhibited a mild limp during ambulation, characterized by a flexed knee posture.

**Figure 1:**
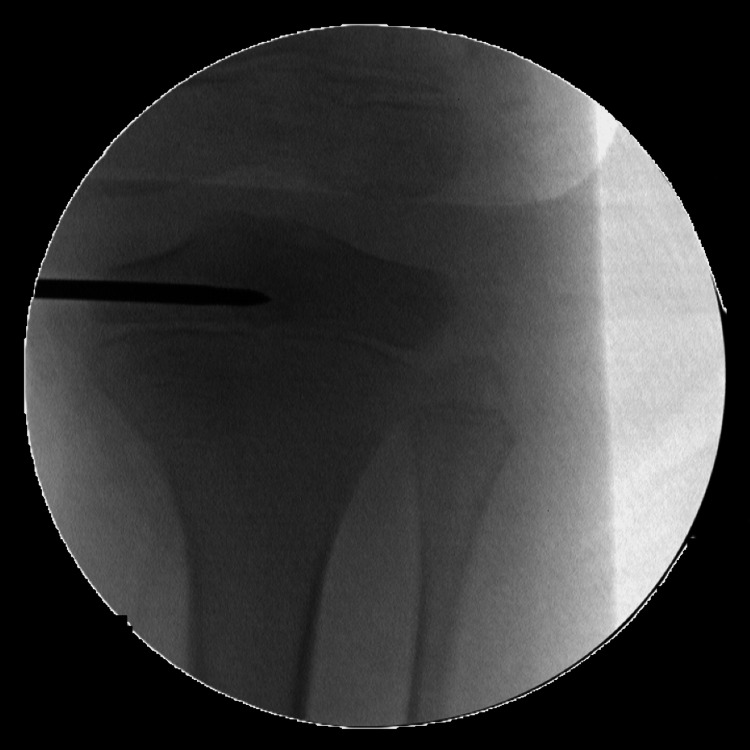
anteroposterior intraoperative radiograph showing the guide wire tip located at the lesion (first surgery)

**Figure 2:**
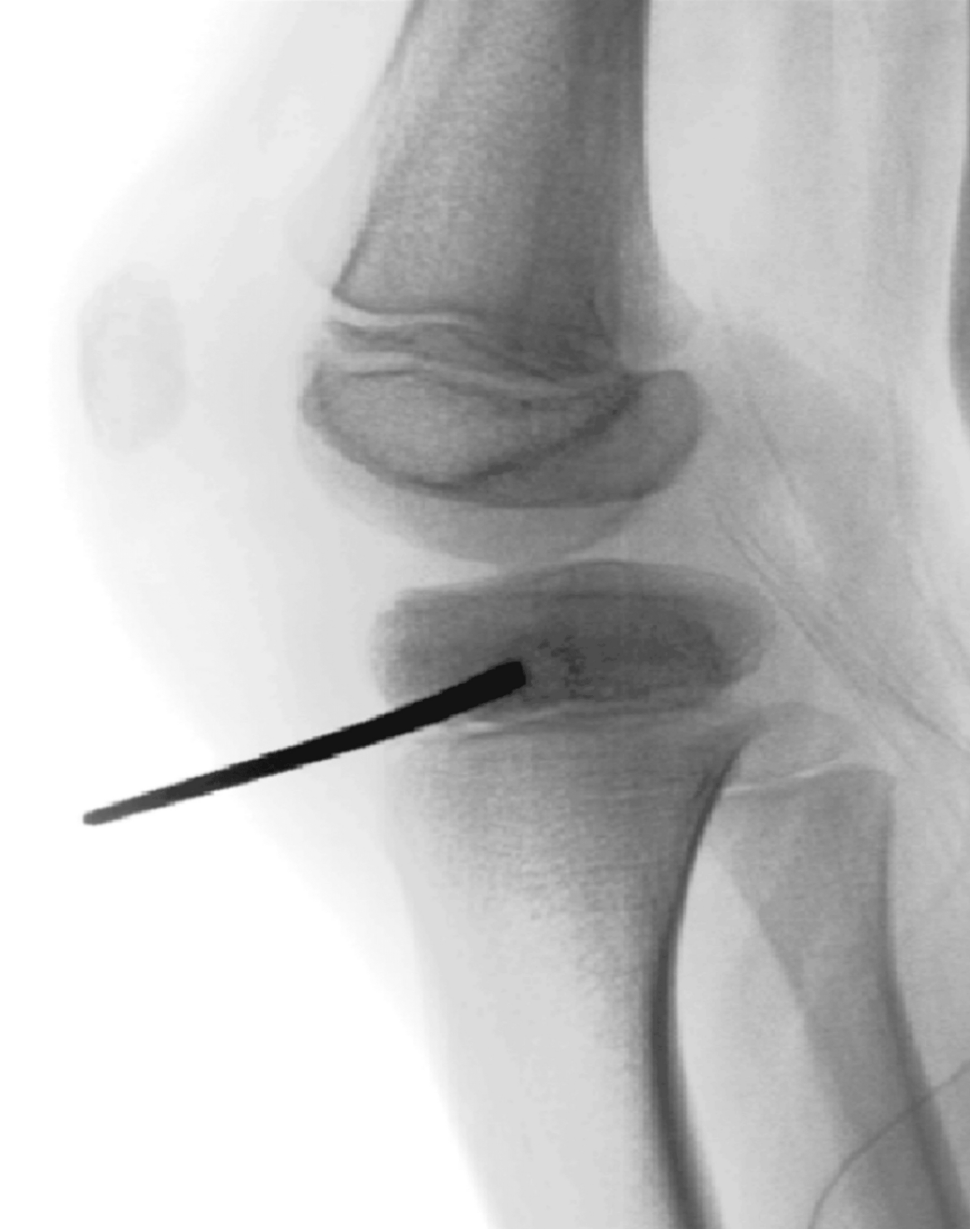
Lateral intraoperative radiograph showing the guide wire tip located at the lesion (first surgery)

Plain radiographs showed localized increased opacity in the proximal tibial epiphysis, just proximal to the growth plate in the center of the epiphysis. The lesion was just adjacent to the epiphysis. MRI showed a localized lesion indicating Brodie’s abscess in the epiphysis. Debridement of the lesion was done through a regular epiphyseal approach (Figures 1-2).

**Figure 3:**
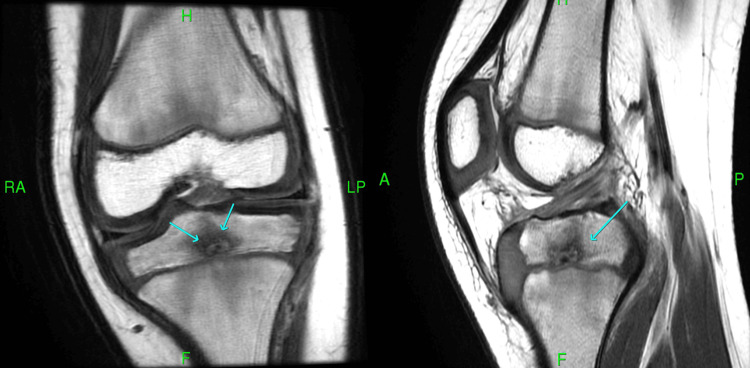
Coronal and sagittal T1-weighted MRI images showing persistence of the lesion (arrows) MRI: magnetic resonance imaging

A good angle for the debridement was not achieved during the initial surgery. Six months after this first debridement, the patient continued to have pain with minimal improvement. A second MRI was done, which showed the persistence of the lesion (Figure 3).

**Figure 4:**
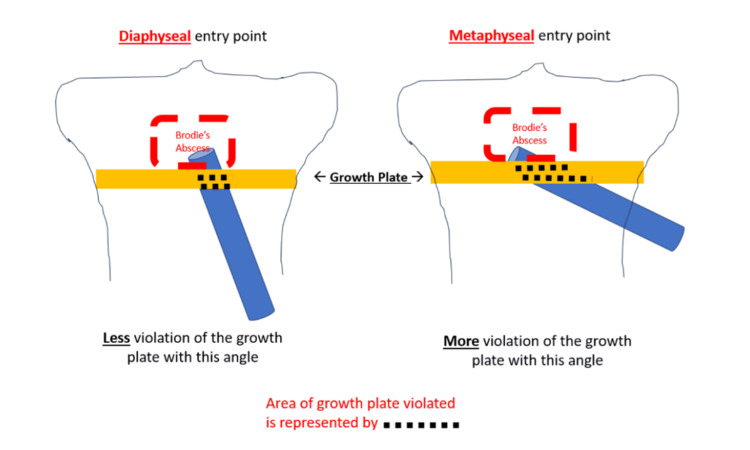
Impact of guide wire entry point (diaphyseal vs. metaphyseal) effect on growth plate violation Image credit and copyright belong to Dr. Ahmed H. Elhessy

Based on that, we opted to proceed with another debridement. Due to the failure of the first debridement from epiphysis, the decision was made to “directly” debride the lesion. The only method to directly debride the lesion was to approach Brodie's abscess from distal to proximal. However, this would entail going through the physis. To decrease the area of the physis violated by drilling, the distal diaphyseal entry point was chosen to approach the abscess rather than approaching it from the metaphysis. This approach aimed to successfully debride the abscess with minimal violation of the growth plate (Figure 4).

Surgical technique

**Figure 5:**
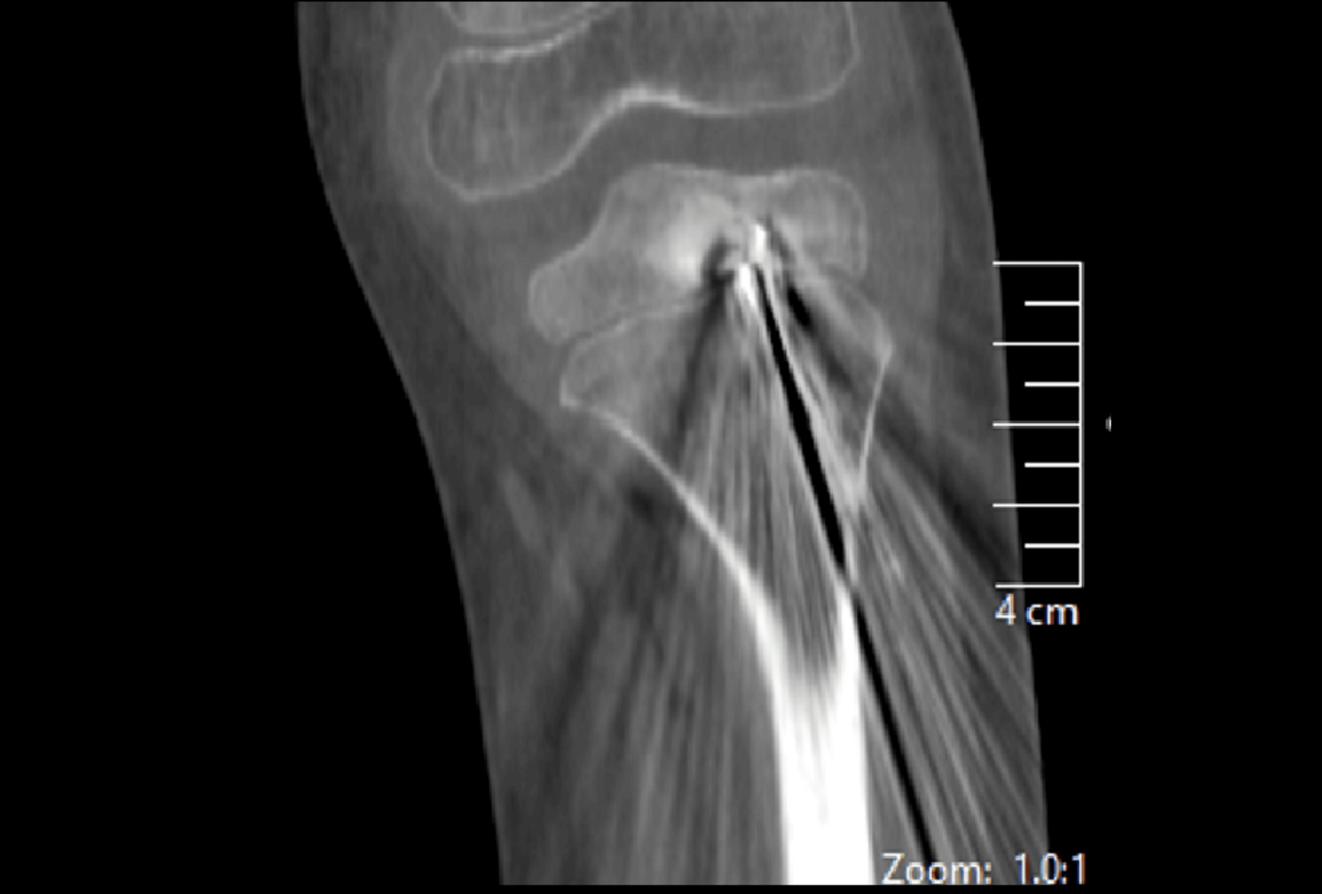
Intraoperative CT coronal image showing the guide wire tip centered in the lesion CT: computed tomography

The position of the abscess was localized intraoperatively based on the MRI study shown previously in Figure 3. Under the guidance of an image intensifier, two 2.8 mm guide wires were introduced into the lesion from the distal tibial diaphysis, one from the medial side and the other from the lateral side. The diaphysis starting point (rather than a metaphyseal starting point) was used to decrease the surface area violated in the growth plate by the guide wires under fluoroscopy guidance. Intraoperative 3D imaging (using the O-arm) was utilized to assess the position of the two guide wires with regard to the lesion. The goal behind positioning two wires within the lesion prior to the usage of the O arm was to precisely locate the lesion with minimal possible radiation exposure to the patient. The lateral wire was found to be precisely in the center of the lesion (Figure 5).

**Figure 6:**
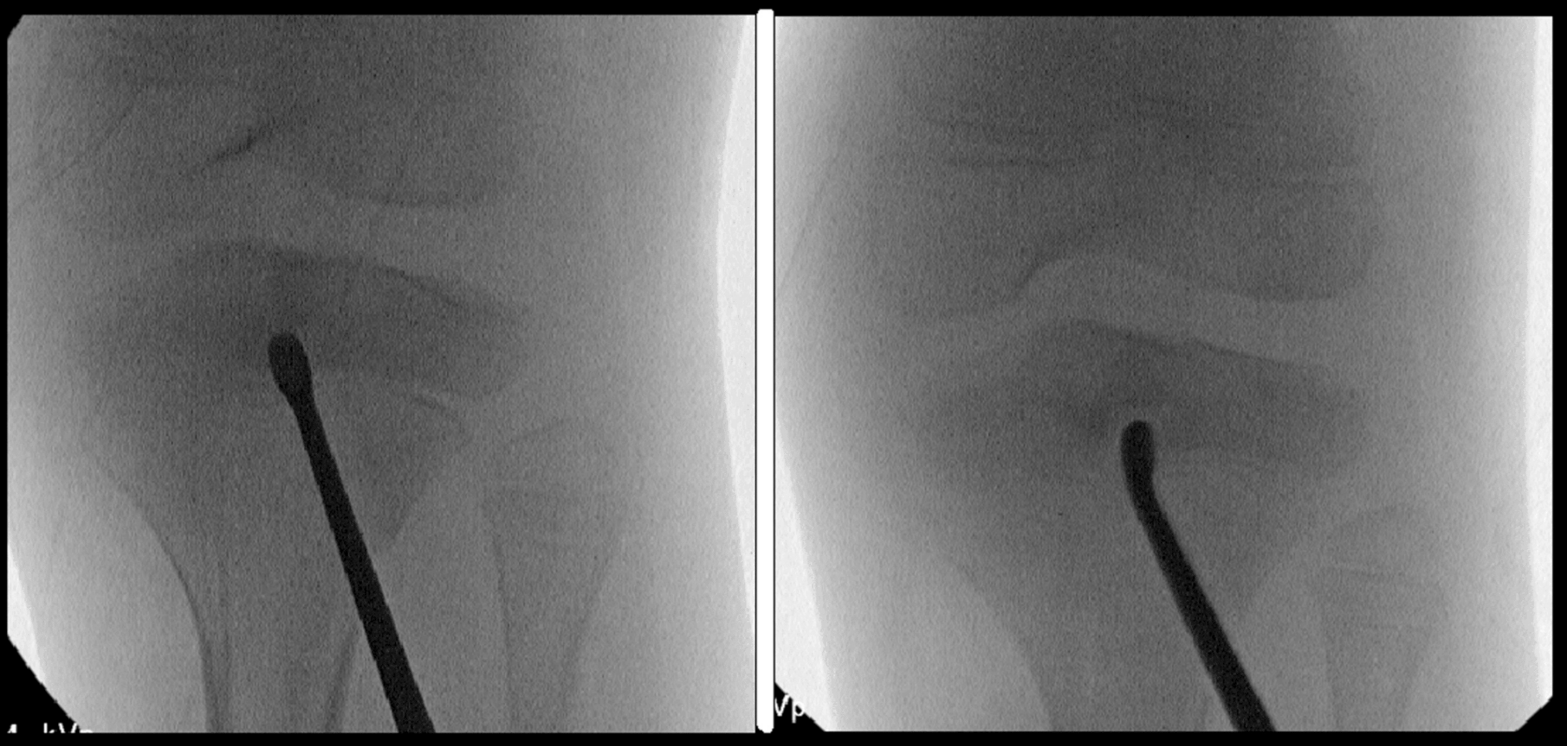
Intraoperative fluoroscopy images showing the curettage of the abscess location with the curved curette at different orientations. A 360-degree curettage was performed throughout the abscess to ensure complete evacuation

After position confirmation, a 4 mm cannulated drill bit was utilized over the lateral guide wire to create a bone tunnel to the lesion. An angled curette was passed through the tunnel to curettage the lesion. A 360-degree curettage was performed throughout the abscess to ensure complete evacuation (Figure 6).

**Figure 7:**
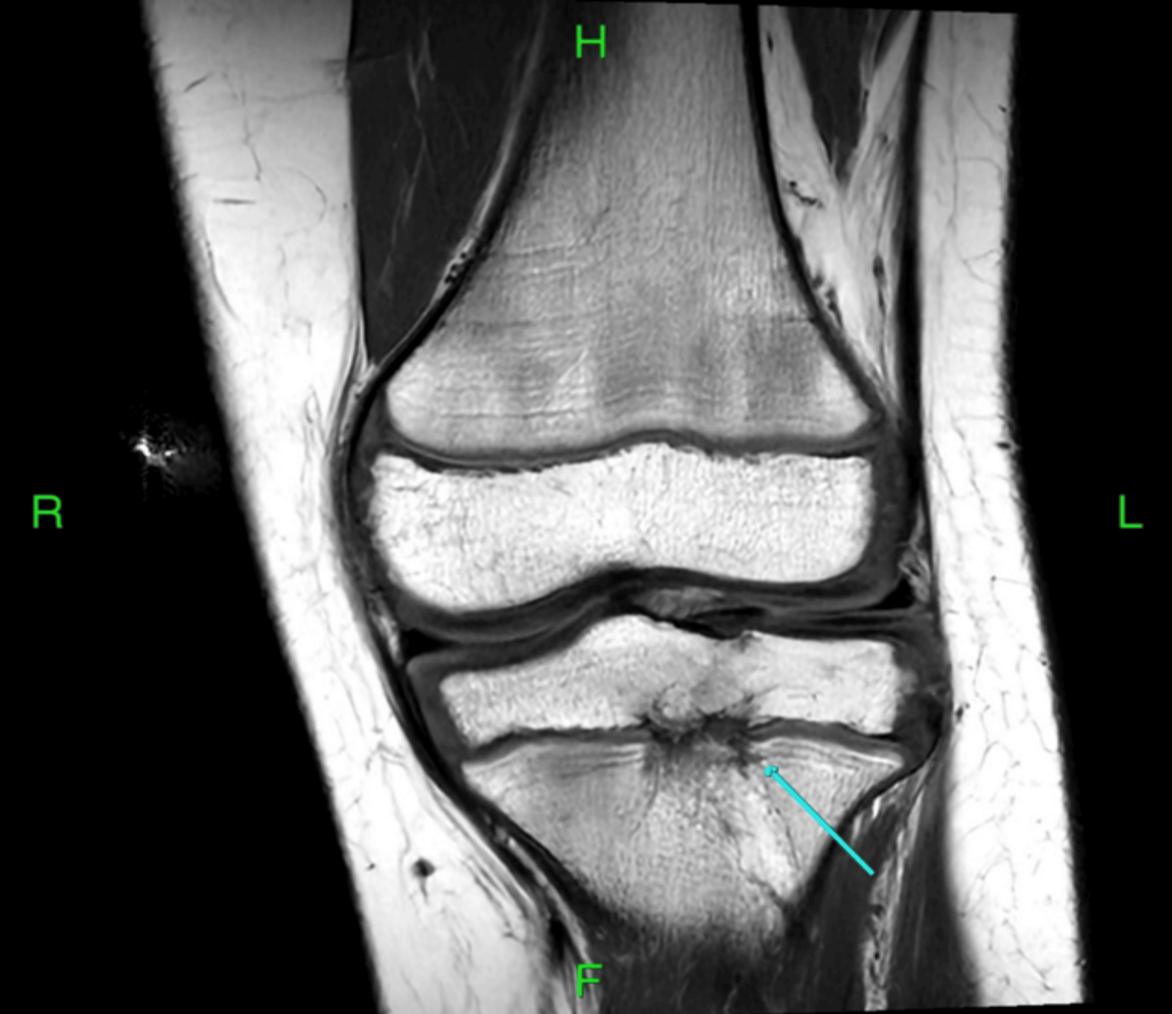
Coronal T2-weighted MRI image showing the absence of the lesion, verifying complete resection of the abscess MRI: magnetic resonance imaging

Postoperatively, the patient was allowed to bear weight immediately as tolerated. During early follow-up visits, the patient's pain improved, and his clinical examination revealed improvement in his knee range of motion and absence of limping on ambulation. Also, his plain knee radiographs showed resolution of the opacity. A repeat MRI (the third MRI for this patient) showed the disappearance of the lesion (Figure 7).

**Figure 8:**
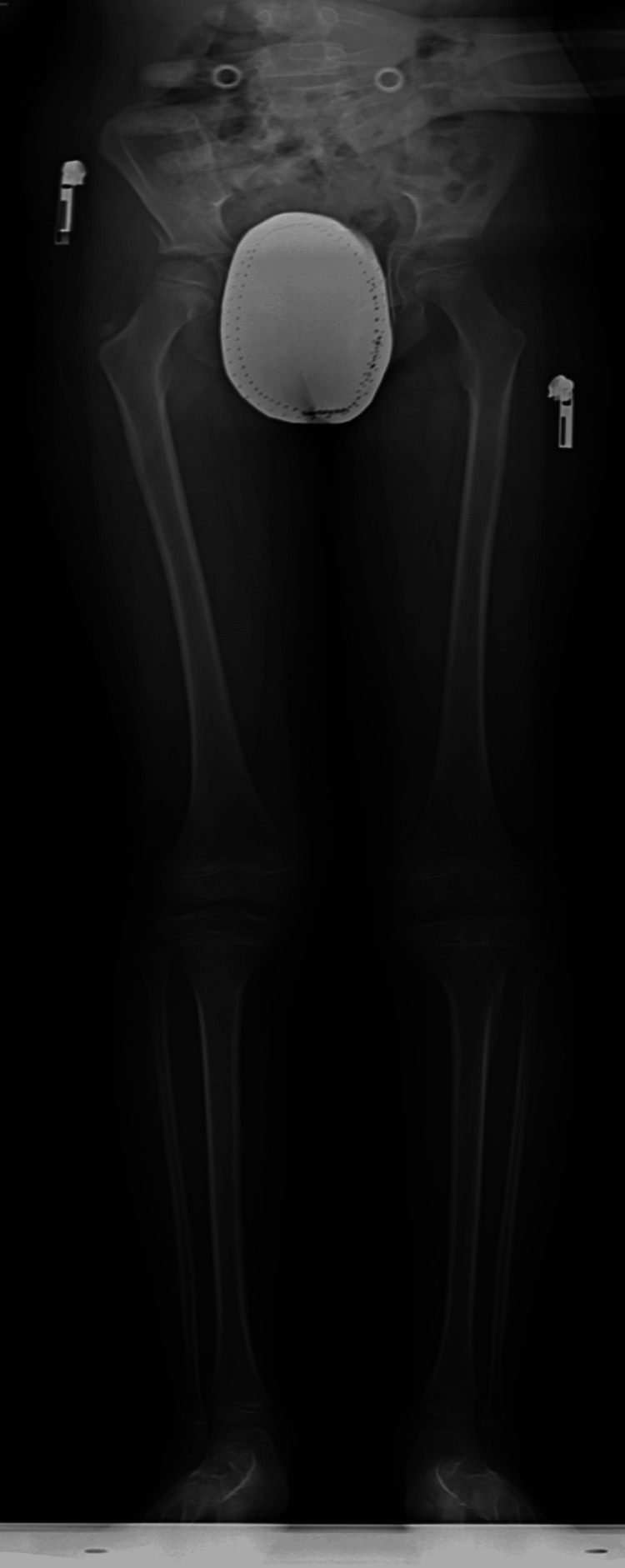
Final follow-up anteroposterior plain radiograph showing no lesion with no residual limb length deformity

A scanogram study done at a one-year follow-up after the second debridement did not show any deformity or shortening. Radiographs at the two-year follow-up revealed no physeal pars or deformity (Figure 8).

## Discussion

Epiphyseal lesions are less common than metaphyseal ones. Differential diagnosis in children includes tumors (e.g., chondroblastoma) or infection (e.g., Brodie's abscess). Green et al. reported eight children diagnosed with subacute osteomyelitis of the femoral or tibial epiphysis. The only presenting complaints were pain and limp. All patients were successfully treated with curettage and oxacillin, with no evidence of damage to the physis after a follow-up period of two to eight years [14]. Bogoch et al. reported the outcomes of the treatment of six children with Brodie’s abscess that traversed the epiphyseal plate. Four were treated with antibiotics and surgical evacuation of their subsequent abscesses, and the remaining two were treated only with antibiotics. None of the patients in their series developed growth arrest [13].

Shah et al. reported successfully treating a subacute physical abscess in a 14-year-old boy with a transphyseal minimally invasive incision and drainage technique [12]. The reported patient also presented after one year of symptom development, which is consistent with our case. The patient showed clinical improvement at a six-month follow-up and had complete remission with no limb length discrepancy during a two-year follow-up period [12].

Transphyseal curettage involves accessing the growth plate responsible for bone growth in the skeletally immature population [15]. Any intervention around this region risks impacting normal bone growth [16]. Surgical treatment aims to debride the abscess via direct curettage. Still, the surgery can potentially disrupt the growth plate and result in complications related to growth disturbance, such as angular deformities and limb length discrepancies [17].

To mitigate the risks associated with Brodie’s abscess curettage, surgeons consider various factors, such as the location of the abscess, the extent of the procedure, and the patient's age. Our approach can serve in treating such cases where physis violation remains inevitable. The narrow trajectory angle can minimize the damage to the affected physis, while the abscess can be drained (Figure 1). We encourage the usage of 3D imaging (O-arm) guidance to locate the center of the lesion. Also, the surgeon’s expertise is essential in determining the lesion location with the minimal number of wire insertions to avoid unnecessary violation of the physis.

Finally, surgical debridement via transphyseal curettage remains an essential pillar in the treatment of Brodie’s abscess. Growth arrest associated with surgery carries the devastating complication of physeal growth arrest, which can affect function, comfort, cosmesis, and quality of life, regardless of the degree of deformity [18]. Orthopedic surgeons must pay attention to performing the required extent of debridement with the least violation to the involved physis to minimize the possibility of growth arrest.

## Conclusions

The literature suggests that epiphyseal Brodie's abscess is a rare but treatable condition. Early diagnosis and treatment are crucial for minimizing the risk of long-term complications. The prognosis for patients with timely and appropriate intervention is generally favorable, highlighting the importance of awareness and prompt clinical response among healthcare providers.

## References

[1] van der Naald N, Smeeing DP, Houwert RM, Hietbrink F, Govaert GA, van der Velde D (2019). Brodie’s abscess: a systematic review of reported cases. J Bone Jt Infect.

[2] Harris NH, Kirkaldy-Willis WH (1965). PRIMARY subacute pyogenıc osteomyelıtıs. J Bone Joint Surg Br.

[3] Lindsetmo RO, Due J, Singh K, Stalsberg H (1993). Brodie's abscess (Article in Norwegian). Tidsskr Nor Laegeforen.

[4] Salik M, Mir MH, Philip D, Verma S (2021). Brodie's abscess: a diagnostic conundrum. Cureus.

[5] Gulati Y, Maheshwari AV (2007). Brodie's abscess of the femoral neck simulating osteoid osteoma. Acta Orthop Belg.

[6] Lowe J, Bridwell RE, Matlock AG, Cibrario A, Oliver J (2020). A case of Brodie’s abscess with tibial erosion and extravasation into surrounding soft tissue. Cureus.

[7] Qi R, Colmegna I (2017). Brodie abscess. CMAJ.

[8] Chin J, Naito T, Hon K, Lomiguen C (2020). Challenges in the diagnosis of Brodie’s abscess in subacute osteomyelitis. J Orthop Case Rep.

[9] El-Feky M, Gaillard F (2023). Brodie Abscess. Radiopaedia.org.

[10] Hourston GJ, Kankam HK, Mitchell PD, Latimer MD (2017). Brodie abscess of the femoral capital epiphysis in a 2-year-old child caused by Kingella kingae. BMJ Case Rep.

[11] Olasinde AA, Oluwadiya KS, Adegbehingbe OO (2011). Treatment of Brodie's abscess: excellent results from curettage, bone grafting and antibiotics. Singapore Med J.

[12] Shah TT, Chin KF, Noorani A, Nairn D (2012). Subacute physeal abscess: case report to illustrate treatment with a minimally invasive incision and drainage technique. Ann R Coll Surg Engl.

[13] Bogoch E, Thompson G, Salter RB (1984). Foci of chronic circumscribed osteomyelitis (Brodie's abscess) that traverse the epiphyseal plate. J Pediatr Orthop.

[14] Green NE, Beauchamp RD, Griffin PP (1981). Primary subacute epiphyseal osteomyelitis. J Bone Joint Surg Am.

[15] Singh V, Garg V, Parikh SN (2021). Management of physeal fractures: a review article. Indian J Orthop.

[16] Dodwell ER, Kelley SP ( 2011). Physeal fractures: basic science, assessment and acute management. Orthop Trauma.

[17] Fury MS, Paschos NK, Fabricant PD (2022). Assessment of skeletal maturity and postoperative growth disturbance after anterior cruciate ligament reconstruction in skeletally immature patients: a systematic review. Am J Sports Med.

[18] Heath MR, Shin TJ, Mehta R (2021). Patients with lower limb deformity report worse quality of life than control subjects regardless of degree of deformity. J Am Acad Orthop Surg Glob Res Rev.

